# Plasticizer Effects in the PVC Membrane of the Dibasic Phosphate Selective Electrode

**DOI:** 10.3390/chemosensors3040284

**Published:** 2015-12-11

**Authors:** Clifton Carey

**Affiliations:** Department of Craniofacial Biology, School of Dental Medicine, University of Colorado, 12800 E. 19th Avenue, MS 8310, Aurora, CO 80206, USA; Tel.: +1-303-724-1046

**Keywords:** ion-selective electrode, chemical sensor, phosphate, plasticizer, lipophilicity, liquid membrane, sensitivity, selectivity

## Abstract

The PVC membrane of an ion-selective electrode (ISE) sensitive to dibasic phosphate ions (HPO_4_-ISE) has not been optimized for maximum selectivity, sensitivity, and useable ISE lifetime and further work was necessary to improve its performance. Two areas of investigation are reported here: include the parameters for the lipophilicity of the plasticizer compound used and the amount of cyclic polyamine ionophore incorporated in the PVC membrane. Six candidate plasticizers with a range of lipophilicity were evaluated for their effect on the useable lifetime, sensitivity, and selectivity of the ISE against 13 different anions. Selectivity was determined by a modified fixed interferent method, sensitivity was determined without interferents, and the usable lifetime evaluated at the elapsed time where 50% of the HPO_4_-ISE failed (L_50_). The results show that choosing a plasticizer that has a lipophilicity similar to the ionophore's results in the best selectivity and sensitivity and the longest L_50_.

## 1. Introduction

An ion-selective electrode sensitive to dibasic phosphate ions (HPO_4_-ISE) that is based on a cyclic polyamine for the ionophore has been previously described [1]. The PVC-based membrane of this sensor was not optimized and further work was necessary to improve its performance. Four areas of investigation were necessary: the plasticizer used in the PVC membrane, the amount of ionophore to be incorporated, the type of support polymer, and the inclusion of ion exchange sites in the form of lipophilic salts. This contribution describes the optimization of the HPO_4_-ISE membrane via the plasticizer and amount of ionophore incorporated into the membrane.

The plasticizer constitutes roughly two thirds of the PVC membrane of the sensor. The ideal plasticizer should have the following properties: softens the PVC membrane, solubilizes the ionophore, insoluble in water, and is inert with respect to ion exchange. Unfortunately, the reality is that while the plasticizer often provides softening, helps solubilize the ionophore, and is insoluble in water, it does not prevent the loss of ionophore to the sample [2]. Additionally, it usually is not completely inert and contributes to the exchange of interfering ions across the membrane [3]. Although the ion exchange rate of the plasticizer is usually small compared to that of the ionophore, the amount of plasticizer relative to the ionophore in the membrane can lead to a significant interfering effect, limiting the sensitivity of the ISE [2,4,5]. Dinten *et al.* have reported that the selectivity and useable lifetime of the ISE is related to the choice and proportion of plasticizer used in the PVC membrane [6]. Current thinking on the appropriate plasticizer to use in the sensor membrane specifies that the lipophilicity of the plasticizer should be as close as possible to that of the ionophore. When the lipophilicities of these two components are matched, I theorize that the plasticizer will be able to dissolve the maximum amount of ionophore, thus providing the most ion binding sites possible. The purpose of this experiment was to investigate plasticizer lipophilicity (*K*_lipo_) and the amount of ionophore incorporated into the membrane as they relate to the useable lifetime, selectivity, and sensitivity of the HPO_4_-ISE. To investigate these effects the sensor membrane components were varied by amount of ionophore and the type of plasticizer used while maintaining the same polymer support (PVC) in the sensor membrane without any lipophilic salts. Six different plasticizers with a range of log*K*_Lipo_ from −0.6 to 6.9 were evaluated for selectivity (*K*_HPO4,j_) to 13 different anions. I hypothesize that there is an optimum plasticizer lipophilicity and ionophore concentration in the sensor membrane to yield the best sensitivity, selectivity, and useable lifetime for the HPO_4_-ISE.

## 2. Experimental Section

### 2.1. Reagents

The following compounds were evaluated as plasticizers in the sensor membrane: *N*,*N*-dimethyformamide (DMF), 2-nitrophenyloctyl ether (NPOE), dibutylphthalate (DBP), tris(2-ethylhexyl)phosphate (TEHP), dibutylsebacate (DBS), and bis(2-ethylhexyl)sebacate (BEHS) were all used as purchased (Fluka Chemical Corp., Ronkonoma, NY, USA). High molecular weight poly(vinyl chloride) (Aldrich Chemical Co., Milwaukee, WI, USA) was used for all membranes. K_2_HPO_4_, 1.00 mol/L KOH, pH standards, NaCl, KNO_3_, KI, NaF, KBr, K_2_SO_4_, KSCN, sodium citrate, potassium acetate, and lithium lactate (Fisher Scientific, Inc., Cincinnati, OH, USA), and buffers HEPES, Bis-Tris, Bis-Tris propane, and potassium phthalate (Aldrich Chemical Co.) were used as received. The ionophore [9-decylhexahydro-1,5,8-triazacyclodecane-2,4-dione, CAS #147150-88-7] was synthesized as described previously [1].

### 2.2. Sensor Fabrication

Membrane-forming cocktails were made by combining varying amounts of plasticizer (600 to 660 mg), PVC (300 to 330 mg), and ionophore (10 to 100 mg) in 5 mL of tetrahydrofuran (THF) and stirring overnight. Note that although the PVC dissolves completely, the ionophore does not completely dissolve by the next morning. Membranes were made directly on the electrode body tip as described earlier [1]: sensor bodies with a tip of Tygon® tubing were dipped into the cocktail solution once and allowed to air dry for 24 h at room temperature. The sensor bodies were then filled with 50 mmol/L potassium phosphate buffer at pH 7.0 that had been spiked with 1 mmol/L potassium chloride and allowed to soak in the same solution for 24 h before testing. Sensor bodies that leaked internal fill solution were discarded. Each sensor was then fitted with an Ag/AgCl half cell and connected to the high side of an electrometer. Up to six experimental sensors and one combination pH electrode (Innovative Sensors, Inc., Anaheim, CA, USA) were connected to a multichannel electrometer (Model MS-314, Elchema, Potsdam, NY, USA) for simultaneous measurements. The Ag/AgCl reference electrode of the combination pH electrode was used as a common reference for all sensors under investigation. Therefore, the electrochemical cell was: Ag|AgCl|KCl_(1 mmol/L)_ + 50 mmol/L phosphate buffer (pH 7.0)‖membrane‖sample solution|KCl_(Sat)_|AgCl|Ag

A minimum of five sensors of each combination were evaluated at room temperature (22 °C). The sensor response was collected via an A/D board in a PC computer with software developed (in house) for these experiments. Millivolt readings of the sensor responses were taken every 100 milliseconds and recorded on disc. Average readings after the sensor had reached equilibrium were then made from at least 600 data points (1 min). For these measurements equilibrium was defined as less than ±0.3 mV change over a minute. The maximum resolution of this A/D system is 0.05 μV. The equilibrium data thus obtained was then imported into a standard spreadsheet for further calculations.

### 2.3. Calculation of Lipophilicity

Lipophilicity (*K*_Lipo_) is defined as the affinity of a molecule for a lipophilic environment and is measured as the partition coefficient in 1-octanal/water [7]. *K*_Lipo_ is used here as the distribution of the compound dissolved in an *n*-octanol and water mixture. This quantity can be measured via standard chromatography techniques or calculated from empirical observations via molecular modeling software. The calculation method is more convenient as the values calculated are all based on the same assumptions and do not include any inherent differences in the compounds that would cause errors in the chromatographic analyses. Additionally published values of *K*_Lipo_ for the plasticizers tested are not consistent. Therefore, the plasticizers and the ionophore were modeled on the Oxford Molecular Modeling system (Beaverton, OR, USA) and the lipophilicity was calculated for each.

### 2.4. Determination of Sensor Sensitivity

**Figure 1:**
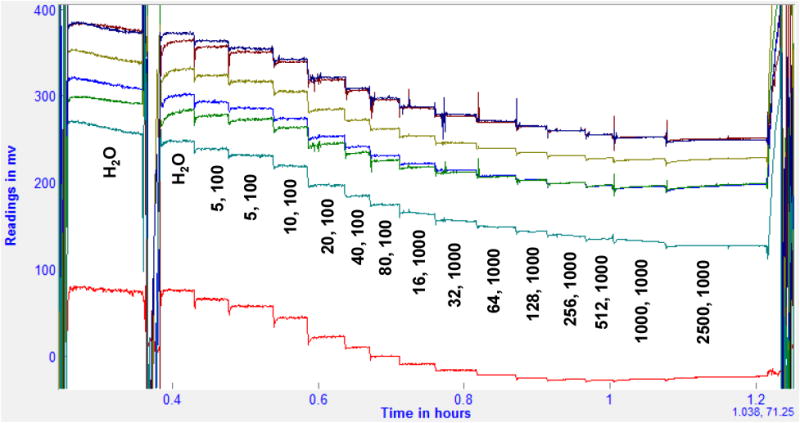
Plot of responses during phosphate additions for a pH and six HPO_4_^2−^ ISEs.

A standard addition method was used to evaluate sensor responses. Sensors were placed in distilled H_2_O and allowed to equilibrate for 20 min before experiments. The sensors along with a combination pH electrode were then placed in 100 mL of distilled H_2_O with a stirrer and allowed to equilibrate until a pseudo-equilibrium was achieved in the potential, usually after 30 min. Then known volumes of concentrated standard (KH_2_PO_4_ titrated to pH 7.2 with KOH) were then added, allowing for equilibration between additions. Figure 1 shows an example of the ISE potentials during phosphate additions for a pH-ISE and six different HPO_4_-ISEs that are tested simultaneously. In this case the six HPO_4_^−2^ ISEs are of the same composition, containing DBP as the plasticizer. The reason that there are different mV potentials between the ISEs is that there is a difference in the membrane thickness between these ISEs resulting in different *E*_0_ values. In most experiments ISEs with a variety of plasticizers are tested at the same time. Table 1 shows the amount of total phosphate standard added at each equilibrium and the calculated HPO_4_^2−^ activity at pH 7.2. Because the pH was measured simultaneously and the calculation of the {HPO_4_^2−^} is based on the pH, no attempt to titrate the sample for any drift in the pH was needed. In this way the standard curves can be determined while eliminating the possibility of contamination between solutions by carry-over. Calculation of the phosphate species activities in solution as a function of the measured pH was done as described earlier [1]. Limit of detection was calculated for the HPO_4_-ISEs by use of the method described by the IUPAC [8]. This method identifies the mean detection limit as the activity of the HPO_4_^2−^species at the intersection of the extrapolation of the ISE's linear response and the null response (where the measured potential does not change). When evaluating the limit of detection where the limit is at a lower concentration than the first phosphate addition for a particular membrane composition, the amount of phosphate was reduced by a factor of 10 to allow for the increased sensitivity. Linear range is reported as the number of decades where the mV response curve has a coefficient of determination (*r*^2^) ≥0.95.

The red curve is the pH electrode potential and the other curves are six different HPO_4_^−2^ ISE potentials of the same membrane composition. The vertical numbers are the volume (μL) and concentration (mmol/L) of phosphate at pH 7.2 added to 100 mL DI-H_2_O.

The phosphate addition schedule is set at pH 7.2 for these experiments. The activities for H_2_PO_4_^−^ and HPO_4_^2−^ species are calculated by use of the Davies modification of the Debye–Hückel Equation [9] from the pH, ionic strength, pKa values for phosphoric acid, the Debye–Hückel constant A = 0.51155 and the Davies constant C = 0.3.

### 2.5. Determination of Selectivity

The selectivity of the sensor for HPO_4_^2−^ ions in the presence of interferent anions (*K*_HPO4,j_) was determined by the fixed interferent method [10] at 1 mmol/L and 10 mmol/L (modified for ionic charge differences) [1]. The following ions were evaluated for their degree of interference: Cl^−^ (NaCl), NO_3_^−^ (KNO_3_), I^−^ (KI), ^−^ (NaF), Br^−^ (KBr), Citrate^3−^ (Na_3_Citrate), SO_4_^2−^ (K_2_SO_4_), SCN^−^ (KSCN), Acetate^−^ (KAcetate), and Lactate^−^ (LiLactate). Additionally, the following buffers were evaluated: 1,3-bis[tris(hydroxymethyl)-methylamino]propane (BTP^−^, neutralized to pH 7.2 with KOH), 2-(bis(2-hydroxyethyl)imino-2-(hydroxymethyl)-1,3-propanediol (B-T^−^, neutralized to pH 6.5 with KOH), phthalate (PHTH^2−^, titrated to pH 5.7 with KOH), and 4-(2-hydroxyethyl)-1-piperazine ethanesulfonate (HEPES^−^, neutralized to pH 7.2 with KOH).

The sensors were placed in distilled H_2_O to soak for 20 min prior to experiments. The sensors were then gently dried and placed in 100 mL of background interferent solution and allowed to equilibrate. The standard addition method described above was used to evaluate the sensor responses. Any changes in the pH were measured simultaneously and no attempt was made to adjust the pH during these experiments. This standard addition method caused the concentration of the background interferent in solution to be slightly reduced as standard was added; however, this reduction was accounted for in the calculations and not significant for the determination of selectivity or sensitivity. Calculations for the selectivity of the membrane were done as described by Carey [1] for each membrane at each interferent concentration. *K*_HPO4,j_ values were then averaged between membranes of the same composition. All sensors evaluated were used for all interferents; that is, if a sensor failed (usually due to leakage of the membrane) in any of the experiments its data was omitted from the averages presented.

### 2.6. Useable Sensor Life Span (L50) Evaluation

A set of membrane-forming cocktails that contained DBP 620 to 670 mg, PVC 330 mg, and 0, 5, 10, 20, or 50 mg of ionophore in 5 mL THF were prepared and stirred for 24 h. Sensor tips were prepared as described above. These sensors were evaluated with the standard addition method described above for response to HPO_4_^2−^ with a 1 mmol/L KCl background daily for the first week and then monthly. The sensors were stored in 50 mL of phosphate buffer at pH 7.0 between evaluations. Sensors were considered unusable when any of the following occurred: slope became less than 24 mV/activity decade, sensitivity below 10^−5^ mol/L HPO_4_^2−^ was not observed, or response time between the additions of standard solution was greater than 180 s. Four sensors from each cocktail were prepared and tested. The time that two sensors of a group were deemed unusable was used as the L_50_ for that cocktail.

## 3. Results and Discussion

The selectivity, sensitivity, and service lifetime of the HPO_4_-ISE are greatly affected by the composition of the PVC membrane that supports the ionophore.

### 3.1. Sensor Sensitivity

**Figure 2:**
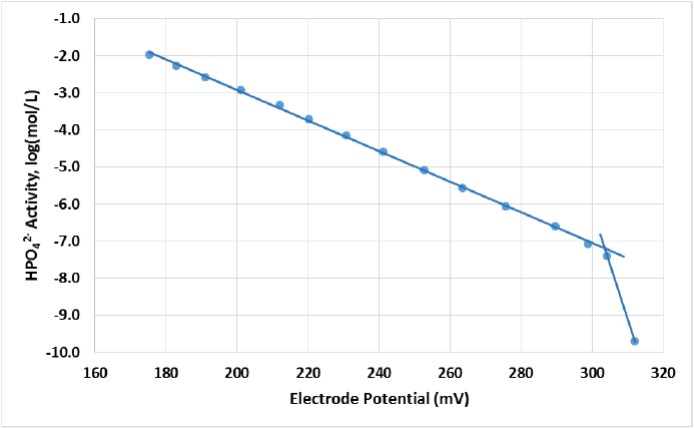
Electrode potential *versus* activity of HPO_4_^2−^ without interferents.

**Figure 3:**
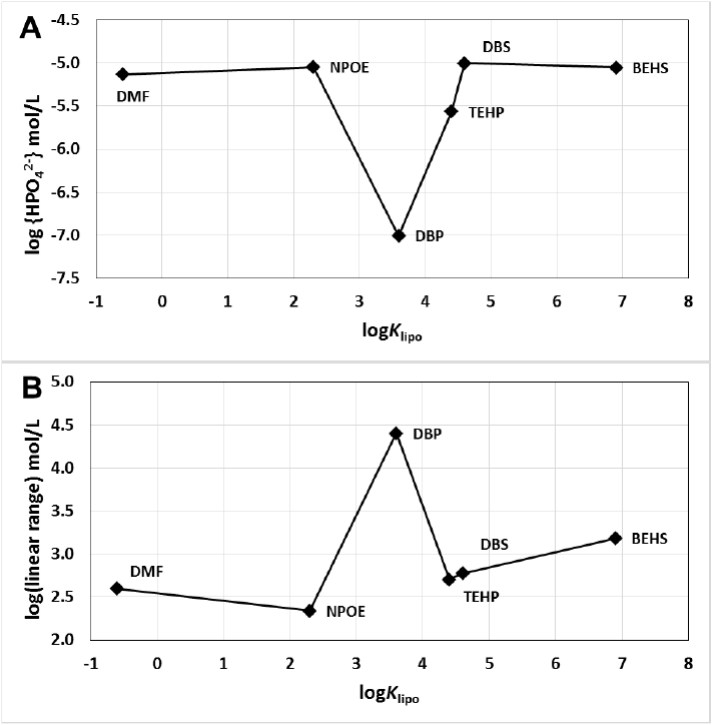
Limit of detection (**A**) and linear range (**B**) as a function of lipophilicity.

Because the limit of detection for HPO_4_-ISEs made with DPB or TEHP was below that of the phosphate addition scheme used for screening the effects of the various plasticizers, these were repeated using a lower amount of phosphate, added such that the additions started at 1.14 × 10^−8^ {HPO_4_^2−^} mol/L at pH 7.2 for ISEs made with DBP or TEHP. An example of the potential response *versus* activity of HPO_4_^2−^ for a fresh ISE made with DBP in the membrane is shown in Figure 2. The average limit of detection and linear range are shown in Figure 3 and Table 2 for the different plasticizers. The best sensitivity was found for the DBP plasticizer, which has a limit of detection at 9.83 × 10^−8^ mol/L {HPO_4_^2−^} and a linear range of 4.85 decades of concentration.

The plot shows the response of a fresh DBP-based ISE to the activity of HPO_4_^2−^ without interferents. The slope is −24.2 mV/concentration decade, the log(detection limit) is −7.19 mol/L, and the linear range is 5.1 concentration decades with an *R*^2^ of 0.9991.

For the limit of detection studies the phosphate addition schedule was modified to allow for lower concentrations of phosphate in the samples. This was required to assess the limit of detection for the HPO_4_-ISE membranes containing DBP and TEHP plasticizers.

### 3.2. Sensor Selectivity

**Figure 4:**
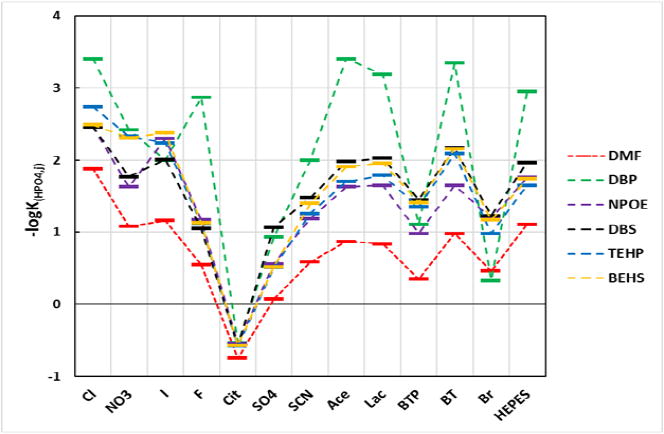
Plasticizer effect on selectivity.

The effects of plasticizer lipophilicity on the HPO_4_-ISE's log*K*_HPO4,j_ against 13 interfering ions are shown in Figure 4 and Table 3. A surprising outcome is that the ISEs responded to citrate ions more than they responded to HPO_4_^2−^ activity in solution. The lipophilicity of the plasticizer used in the membrane had little effect, possibly indicating that the apparent selectivity towards the citrate ion is not necessarily reversible or that the citrate ion is exchanging through the plasticizer rather than the ionophore.

**Figure 5:**
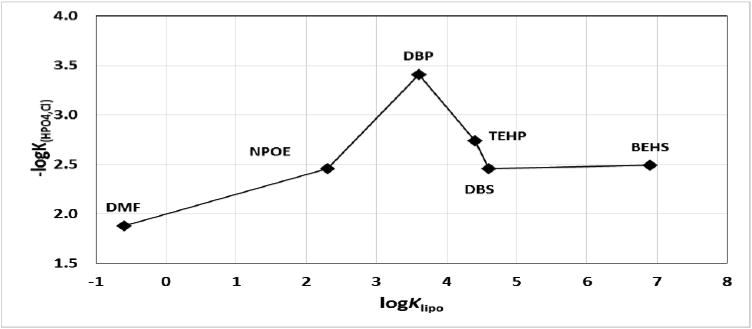
Effect of plasticizer lipophilicity on selectivity against Cl^−^ ion.

Careful evaluation of the data shows that the plasticizer BEHS interacts with interferents in a manner that is similar to the interactions of DBS. Possibly, the affinity of the ester groups in BEHS is similar to those in DBS for the interferents, both of which result in inferior performance characteristics. One could interpret the data presented in this paper in a different way: if the *K*_lipo_ is more than 2 orders of magnitude different from the ionophore, the resulting ISE parameters (selectivity, slope, linear range) will be inferior when compared to an ISE made with a plasticizer of similar *K*_lipo_. The lipophilicity of the plasticizer used in the membrane was a major factor in the selectivity for the other interfering ions. The lipophilicity of the plasticizers DBP and TEHP are closest to that calculated ionophore which has a log*K*_Lipo_ of 3.9. Chloride ions are ubiquitous in the environment and in biological fluids; therefore, an HPO_4_-ISE would need greatest selectivity against Cl^−^ to be of greatest utility. Figure 5 shows the selectivity of the HPO_4_-ISEs made with the various plasticizers to Cl^−^. The ISE membrane prepared with the DBP plasticizer is the best for selectivity against chloride ions.

The selectivity values are the negative logarithms of the selectivity coefficients (−log*K*). DMF = dimethylformamide, NPOE = 2-nitrophenyloctyl ether, DBP = dibutylphthalate, TEHP = Tris(2-ethylhexyl)phosphate, DBS = dibutylsebacate, and BEHS = Bis(2-ethylhexyl)sebacate. *N* ≥ 3 electrodes for each condition; reproducibility = ±0.1 log units. Note that the larger the −log*K*_HPO4,j_ value, the greater the selectivity against the interferent.

The selectivity of HPO4-ISE membranes with the DBP plasticizer was superior to all other plasticizer membranes for all anions except for I−, Br−, SO_4_^2−^, and the BTP^−^ (bis[tris(hydroxymethyl)-methylamino]propane) buffer system. While a mechanistic explanation for why these interfering ions with the HPO_4_-ISE at optimal composition (35 mass % PVC, 60 mass % DBS, and 5 mass % ionophore) has not been proven, initial experiments show that when the HPO_4_-ISE fill solution contains an amount of these interferents similar to that present in the external sample then the HPO_4_-ISE tends to have a slightly depressed linear range and a shorter useable service life yet retains selectivity for the HPO_4_^2−^ ion. Therefore, a practical approach to attain selectivity in the presence of these interferents is to include the same concentration of the interferent as is present in the sample within the ISE fill solution. This is because the lack of activity across the membrane removes the potential (EMF) across the membrane due to that ion. The useable service life of the HPO_4_^−^ISE is greatly reduced as the concentrations of I^−^, Br^−^, and SO_4_^2−^ are increased in the fill solution due to the formation of Ag-halides or Ag-SO_4_ ion-pairs or precipitates on the internal Ag/AgCl half-cell.

The −log*K*_(HPO4,Cl)_ of the HPO_4_-ISE membranes made with DBP is superior to all other membrane types.

### 3.3. Useable Sensor Life Span (L_50_)

**Figure 6:**
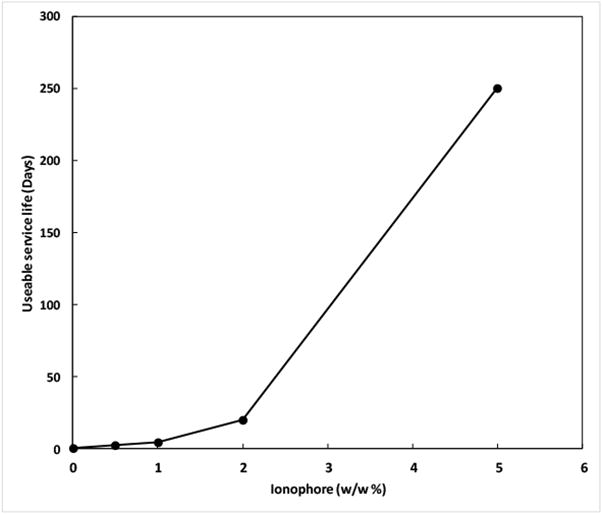
Useable service life of the DBP-PVC based HPO4-ISE with varying amounts of ionophore.

The evaluation of the L_50_ for useable sensor life was based on the concept that the more ionophore that can be entrained into the PVC membrane the longer its useable period will be. The membrane solutions with 0%, 0.5%, 1.0%, 2.0%, and 5.0% (*w*/*w*) ionophore were heated to 40 °C and stirred overnight to enhance dissolution of the ionophore into the DBP-PVC-tetrahydrofuran cocktail. Membranes were formed from these cocktails on electrode bodies at 40 °C for several hours before cooling to room temperature. At 40 °C all the membranes were clear and at room temperature the membranes containing 2% ionophore were hazy and membranes containing 5% ionophore were cloudy. Further evaluation showed that the DBP plasticizer can dissolve approximately 2% of ionophore at room temperature. The particles within the membrane are ionophore, which remains in the membrane and is dissolved as ionophore is lost to the samples. Failed membranes were clear and did not contain any undissolved ionophore particles. The slow loss of ionophore to the sample thus greatly extended the L_50_, as shown in Figure 6.

The L_50_ of the DBP-PVC based HPO4-ISE is extended when greater amounts of ionophore are entrained within the membrane. Approximately 2% (*w*/*w*) of ionophore is soluble in the DBP plasticizer, with the remaining dispersed throughout the membrane as solid particles. These particles dissolve into the membrane to replace ionophore lost to the samples, thus extending the service life of the ISE.

## 4. Conclusions

These results show that the plasticizer in the membrane had a large effect on sensitivity, selectivity, and useable service life. DBP and TEHP exhibited the best selectivity characteristics. The calculated log*K*_Lipo_ values for these two plasticizers are close to that of the ionophore (log*K*_Lipo_ = 3.9), supporting the hypothesis that the lipophilicity of the plasticizer should match the ionophore for achieving optimal ISE performance. The DBP containing ISEs showed sufficient selectivity for typical oral fluid anions (such as Cl^−^, NO_3_^−^, F^−^, and lactate^−^) to allow the direct measurement of phosphate activities in saliva and environmental waters.

## Figures and Tables

**Table 1:** Phosphate additions and the resulting {H_2_PO_4_^−^} and {HPO_4_^2−^} activities at pH 7.2.

#	Added PO_4_ μL	Added PO_4_ mmol/L	Tot Vol	TOT PO_4_ mmol/L	{H_2_PO_4_^−^} mol/L	{HPO_4_^−2^} mol/L	log{H_2_PO_4_}	log{HPO_4_^2−^}
1	0	0	100,000	1.00E–09	6.62E–13	6.77E–13	−12.179	−12.169
2	5	100	100,005	5.00E–03	2.45E–06	2.51E–06	−5.611	−5.601
3	5	100	100,010	1.00E–02	4.88E–06	4.99E–06	−5.312	−5.302
4	10	100	100,020	2.00E–02	9.70E–06	9.93E–06	−5.013	−5.003
5	20	100	100,040	4.00E–02	1.92E–05	1.97E–05	−4.716	−4.706
6	40	100	100,080	7.99E–02	3.81E–05	3.89E–05	−4.420	−4.410
7	80	100	100,160	1.60E–01	7.49E–05	7.66E–05	−4.126	−4.116
8	16	1000	100,176	3.19E–01	1.47E–04	1.50E–04	−3.834	−3.824
9	32	1000	100,208	6.39E–01	2.84E–04	2.91E–04	−3.546	−3.536
10	64	1000	100,272	1.28E+00	5.45E–04	5.57E–04	−3.264	−3.254
11	128	1000	100,400	2.55E+00	1.03E–03	1.05E–03	−2.989	−2.979
12	256	1000	100,656	5.09E+00	1.89E–03	1.93E–03	−2.724	−2.714
13	512	1000	101,168	1.01E+01	3.38E–03	3.46E–03	−2.470	−2.460
14	1000	1000	102,168	1.98E+01	5.82E–03	5.95E–03	−2.235	−2.225
15	2500	1000	104,668	4.32E+01	1.07E–02	1.09E–02	−1.973	−1.963

**Table 2:** Limit of detection, linear sensitivity, and linear slope of HPO_4_^2−^ ISEs with various plasticizers in the membrane.

Plasticizer	log*K*_lipo_	Limit of Detection –log{HPO_4_^2−^} mol/L		Linear Sensitivity Conc. Decades		Linear Slope mV/Conc. Decade	
		
		Average	Std. Dev	Average	Std. Dev	Average	Std. Dev
DMF	−0.6	−5.13	0.38	2.60	0.00	−7.4	0.9
NPOE	2.3	−5.05	0.20	2.34	0.33	−8.8	4.9
DBP	3.6	−7.01	0.18	4.85	0.21	−23.8	0.6
TEHP	4.4	−5.56	0.92	2.70	0.46	−6.7	11.6
DBS	4.6	−5.00	0.51	2.78	0.42	−9.3	2.2
BEHS	6.9	−5.05	0.26	3.18	1.57	−16.0	4.4

**Table 3:** Selectivity coefficients (−log*K*HPO_4,J_) as a function of lipophilicity for 13 interferents.

Plast.	log*K*_Lipo_	Cl^−^	NO3^−^	I^−^	F^−^	Cit_3_^−^	SO_4_^2−^	SCN^−^	Ace^−^	Lac^−^	BTP^−^	B-T^−^	Br^−^	HEPES
DMF	−0.6	1.88	1.08	1.16	0.55	−0.75	0.07	0.59	0.87	0.84	0.35	0.98	0.46	1.11
NPOE	2.3	2.46	1.63	2.30	1.17	−0.55	0.56	1.19	1.63	1.65	0.98	1.65	1.19	1.76
DBP	3.6	3.41	2.42	2.00	2.87	−0.54	0.93	2.00	3.41	3.19	1.11	3.35	0.33	2.95
TEHP	4.4	2.74	2.33	2.24	1.12	−0.58	0.51	1.26	1.70	1.79	1.35	2.09	0.98	1.65
DBS	4.6	2.46	1.77	2.01	1.05	−0.58	1.07	1.48	1.98	2.03	1.45	2.17	1.23	1.97
BEHS	6.9	2.49	2.31	2.38	1.13	−0.57	0.52	1.40	1.91	1.96	1.41	2.16	1.17	1.74
